# How Phagocytic Cells Kill Different Bacteria: a Quantitative Analysis Using Dictyostelium discoideum

**DOI:** 10.1128/mBio.03169-20

**Published:** 2021-02-16

**Authors:** Tania Jauslin, Otmane Lamrabet, Xenia Crespo-Yañez, Anna Marchetti, Imen Ayadi, Estelle Ifrid, Cyril Guilhen, Matthias Leippe, Pierre Cosson

**Affiliations:** a Department of Cell Physiology and Metabolism, Faculty of Medicine, University of Geneva, Geneva, Switzerland; b Zoological Institute, Comparative Immunobiology, University of Kiel, Kiel, Germany; CIML

**Keywords:** *Dictyostelium discoideum*, *Klebsiella pneumoniae*, *Escherichia coli*, *Pseudomonas aeruginosa*, *Staphylococcus aureus*, *Bacillus subtilis*, killing, lysozyme, AlyL

## Abstract

Ingestion and killing of bacteria by phagocytic cells protect the human body against infections. While many mechanisms have been proposed to account for bacterial killing in phagosomes, their relative importance, redundancy, and specificity remain unclear. In this study, we used the Dictyostelium discoideum amoeba as a model phagocyte and quantified the requirement of 11 individual gene products, including nine putative effectors, for the killing of bacteria. This analysis revealed that radically different mechanisms are required to kill Klebsiella pneumoniae, Escherichia coli, Pseudomonas aeruginosa, Staphylococcus aureus, and Bacillus subtilis. AlyL, a lysozyme-like protein equipped with a distinct bacteriolytic region, plays a specific role in the intracellular killing of K. pneumoniae, with assistance from BpiC and Aoah, two lipopolysaccharide (LPS)-binding proteins. Rapid killing of E. coli and P. aeruginosa requires the presence of BpiC and of the NoxA NADPH oxidase. No single effector tested is essential for rapid killing of S. aureus or B. subtilis. Overall, our observations reveal an unsuspected degree of specificity in the elimination of bacteria in phagosomes.

## INTRODUCTION

Phagocytic cells such as macrophages and neutrophils play an essential role in the defense of our body against invading microorganisms. Invading microorganisms are ingested by phagocytic cells and subsequently found in membrane-delimited phagosomes, where they are killed. Numerous gene products directly engaged in bacterial killing inside phagosomes have been identified over several decades. These putative effectors fall into four major categories: ions (including protons), free radicals, digestive enzymes, and membrane-permeabilizing antimicrobial peptides or proteins (1, 2). These four categories can be further refined into subcategories (e.g., digestive enzymes can be glycosidases, lipases, or proteases) and are often narrowed down to specific functional families and gene products (e.g., glycosidases with lysozyme activity) (3). Other gene products are necessary for efficient bacterial killing, but are not directly engaged in this process, such as gene products that ensure maturation of newly formed phagosomes into bacteriolytic organelles, that control phagosomal ionic composition (4), or that participate in signaling pathways regulating intracellular killing in response to extracellular or intracellular signals (5).

Individual studies of the phagocytic process have often focused on a single facet of intracellular killing (e.g., the role of one specific gene product in the killing of one or a few bacterial species). As a consequence, despite the vast accumulated knowledge, many fundamental questions remain unanswered. The relative importance of different mechanisms in bacterial killing has not been established. The degree of redundancy between the different mechanisms remains to be determined. Likewise, it is not clear to what extent phagocytes use different effectors to kill different species of bacteria.

Dictyostelium discoideum is a soil amoeba that feeds by ingesting, killing, and digesting microorganisms. To bind, ingest, and kill bacteria, D. discoideum uses molecular mechanisms analogous to those found in specialized phagocytic cells of multicellular organisms, such as neutrophilic granulocytes or macrophages (6, 7). Due to the relative ease with which haploid D. discoideum cells can be grown, observed, and genetically manipulated, they have been used extensively to discover and analyze the role of specific gene products in various facets of the phagocytic process. One method has been to identify mutants with interesting phenotypic alterations by screening libraries of random mutants. Notably, this method led to the discovery of two gene products that participate indirectly in bacterial killing by D. discoideum: Kil1 (8) and Kil2 (9). Kil1 is a Golgi sulfotransferase involved in the maturation of lysosomal enzymes. Its genetic inactivation leads to defective sulfation of lysosomal enzymes and to a decrease in the ability of phagocytes to kill ingested Klebsiella pneumoniae bacteria (8). Kil2 is a phagosomal P-type ATPase that has been proposed to transport magnesium ions from the cytosol to the phagosomal lumen. Its genetic inactivation causes a decrease in proteolytic activity in phagosomes and in intracellular killing of K. pneumoniae (9). Alternatively, genes encoding putative effectors have been identified by searching the well-annotated D. discoideum genome for orthologues of previously identified effectors such as lysozymes (10).

In this study, we used D. discoideum to assess the differential roles of Kil1, Kil2, nine putative effectors (NoxA, AlyA, AlyL, BpiC, Aoah, AplA, AplN, CtsB, and CtsD), and acidic pH in the intracellular killing of several Gram-negative and Gram-positive bacteria. NoxA, the main superoxide-producing NADPH oxidase in D. discoideum phagosomes (11, 12), and AlyA, the main D. discoideum lysozyme (13, 14), would be expected to play an important role in the killing of many bacteria. The putative role of AlyL, a poorly studied AlyA-like protein upregulated in D. discoideum exposed to Gram-negative bacteria, is more hypothetical (10, 15). Likewise, the putative role of CtsB and CtsD cathepsins (16, 17) remains to be established. Bactericidal permeability-increasing protein (BpiC) binds lipopolysaccharides (LPS) in the cell wall of Gram-negative bacteria, while acyloxyacyl hydrolase (Aoah) removes acyl chains from the lipid A moiety of LPS. Both BpiC and Aoah have been proposed to participate in intracellular killing of bacteria (18, 19). Finally, AplA and AplN, two amoebapore-like proteins may in principle permeabilize bacterial membranes (20).

## RESULTS

### Intracellular killing of different bacteria in mutant phagocytes.

**FIG 1:**
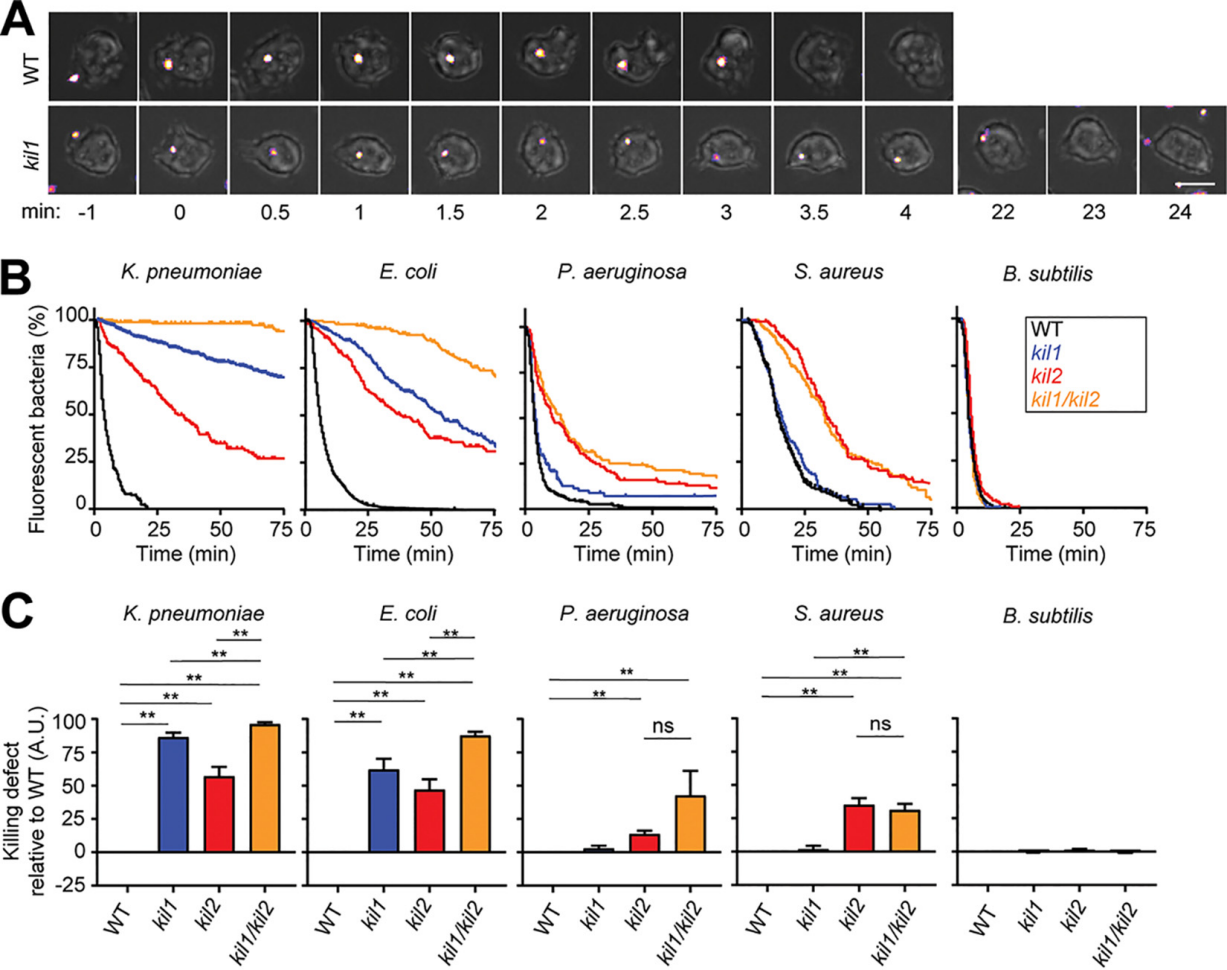
Kil1 and Kil2 are differentially required for the intracellular killing of different bacteria. To visualize ingestion and intracellular killing of individual bacteria, D. discoideum cells were incubated with GFP‐expressing K. pneumoniae, E. coli, P. aeruginosa, S. aureus, or mCherry-expressing B. subtilis at a ratio of 1:3 for 2 h. Cells were imaged every 30 s by phase-contrast and fluorescence microscopy. (A) The top row shows successive images of a WT D. discoideum cell ingesting (*t* = 0) and killing (*t* = 3.5 min) an individual K. pneumoniae bacterium. In the bottom row, a *kil1* KO cell killed a K. pneumoniae bacterium 23 min after ingestion. Scale bar, 10 μm. (B) The time between ingestion and fluorescence extinction was determined for each bacterium, and the probability of remaining fluorescent was represented as a function of time after ingestion. Bacterial killing was analyzed in WT, *kil1* KO, *kil2* KO, and *kil1 kil2* double KO D. discoideum cells. The curves shown were obtained by pooling the results of *n* independent experiments: *n* = 6 for K. pneumoniae and *n* = 5 for E. coli, P. aeruginosa, S. aureus, and B. subtilis. (C) Quantification of the normalized area under the curve (AUC) of independent experiments. In each independent experiment, a WT control was included, and its AUC was subtracted from the AUC determined for each mutant cell before normalization. Values are means ± standard errors of the means (SEM). **, *P* < 0.005, Mann-Whitney test. ns, nonspecific difference; A.U., arbitrary units. The detailed method used to calculate and represent graphically our observations is shown in Fig. S1 in the supplemental material.

In order to determine which cellular mechanisms are necessary for intracellular killing of ingested bacteria, we first measured the ability of the previously characterized *kil1* knockout (KO), *kil2* KO, and *kil1 kil2* double KO D. discoideum mutants to kill five different bacteria. We fed bacteria producing fluorescent proteins to D. discoideum cells and measured for each individual bacterium the time between its ingestion and the extinction of its fluorescence. Extinction of bacterial fluorescence has been previously used by us and others as a proxy for bacterial death (9, 21–23). In this study, we use the term “killing” as synonymous with “extinction of fluorescence.” The representative images shown in Fig. 1A show green fluorescent protein (GFP)-producing K. pneumoniae cells phagocytosed by D. discoideum. In these two representative examples, extinction of GFP fluorescence occurred 3.5 min after ingestion by a wild-type (WT) cell and 23 min after ingestion by a *kil1* KO cell. Notably, in our experience, the time required to kill ingested bacteria was highly dependent on the exact conditions under which both D. discoideum cells and bacteria were grown and on the experimental setup. To allow meaningful comparisons, all cells were grown under similar conditions (cell density and timing), and all experiments presented in this study included a WT control used in parallel with one or several mutants. For each situation analyzed (e.g., killing of ingested K. pneumoniae in *kil1* KO cells), the time required for fluorescence extinction was quantified in at least five independent experiments, and at least 30 phagocytic events were analyzed in each experiment. The observations were then combined and plotted as a Kaplan-Meier curve representing the percentage of fluorescent bacteria as a function of time after ingestion (Fig. 1B). In addition, intracellular killing of bacteria in mutant cells was compared to killing in WT cells in each independent experiment to assess the experimental variability and the significance of the observed results (Fig. 1C). The method used to calculate and represent graphically our observations is detailed in Fig. S1 in the supplemental material. Using this approach, we assessed the intracellular killing of five different bacteria: three Gram negative (K. pneumoniae, Escherichia coli, and Pseudomonas aeruginosa) and two Gram positive (Staphylococcus aureus and Bacillus subtilis). We first measured their intracellular killing in WT, *kil1* KO, *kil2* KO, and *kil1 kil2* double KO D. discoideum cells. We observed that the intracellular killing of K. pneumoniae is almost entirely dependent on Kil1 and Kil2, as evidenced by the fact that *kil1 kil2* double KO cells almost completely failed to kill ingested K. pneumoniae (Fig. 1B and C). Killing of other bacteria showed different requirements: compared to WT cells, the bacterial killing in *kil1 kil2* double KO cells was partially reduced for E. coli, P. aeruginosa, and S. aureus, but not for B. subtilis (Fig. 1B and C). Analysis of single-gene KO mutants revealed additional specific differences: Kil1 played a significant role in the killing of K. pneumoniae and E. coli, but not that of P. aeruginosa, S. aureus, or B. subtilis (Fig. 1B and C). Kil2 was necessary for efficient killing of K. pneumoniae, E. coli, P. aeruginosa, and S. aureus, but not B. subtilis. In order to confirm these observations with a different method, we measured the decrease of bacterial CFU when bacteria were incubated in suspension in the presence of D. discoideum cells. This type of experiment can only detect strong delays in bacterial killing, since phagocytosis of bacteria is relatively slow (a few hours) compared to intracellular killing. In agreement with microscopic observations, K. pneumoniae and E. coli were killed more slowly in *kil1* KO, *kil2* KO, and *kil1 kil2* double KO cells than in WT cells (see Fig. S2A and B in the supplemental material). On the contrary B. subtilis cells were killed as rapidly in *kil1* KO, *kil2* KO, *kil1 kil2* double KO, and WT cells (Fig. S2C). Together, these results indicate that different mechanisms are required for efficient killing of different bacteria in phagosomes.

10.1128/mBio.03169-20.2 FIG S1 Related to Fig. 1. Killing of bacteria by mutant *D. dicoideum* cells: calculation and representation. We provide here the example of how we calculated and represented the destruction of E. coli by kil1 KO cells. (A) For each experiment, we determined the area under the curve (AUC), and we calculated the area shaded in blue (AUC*_kil1_* − AUC_WT_). (B) The whole area of the graph is 7,500 (75 min × 100%). For each bacterium, the maximal ability of WT cells to destroy the bacterium is 7,500 − AUC_WT_. (C) The defect of *kil1* KO cells was calculated by dividing these two numbers (AUC*_kil1_* − AUC_WT_/7,500 − AUC_WT_) to generate a figure comprising between 0% (no defect) and 100% (total loss of bacterial killing). The data used in this figure are taken from Fig. 1. Download FIG S1, TIF file, 1.1 MB. Copyright © 2021 Jauslin et al. 2021 Jauslin et al. https://creativecommons.org/licenses/by/4.0/ This content is distributed under the terms of the Creative Commons Attribution 4.0 International license.

10.1128/mBio.03169-20.3 FIG S2 Related to Fig. 1. Genetic inactivation of *kil1* and *kil2* slows killing of K. pneumoniae and E. coli, but not that of B. subtilis. K. pneumoniae (A), E. coli (B), or B. subtilis (C) cells were mixed with WT, *kil1* KO, *kil2* KO, or *kil1 kil2* double KO D. discoideum cells. At the indicated times, an aliquot of the mixture was collected, D. discoideum cells were lysed, the bacteria were plated on LB agar, and the total (extracellular and intracellular) number of remaining viable bacteria was evaluated by counting colony-forming units (CFU). Results are expressed as a percentage of CFU at time 0 (median ± SEM; *n* = 3). Download FIG S2, TIF file, 0.4 MB. Copyright © 2021 Jauslin et al. 2021 Jauslin et al. https://creativecommons.org/licenses/by/4.0/ This content is distributed under the terms of the Creative Commons Attribution 4.0 International license.

**FIG 2:**
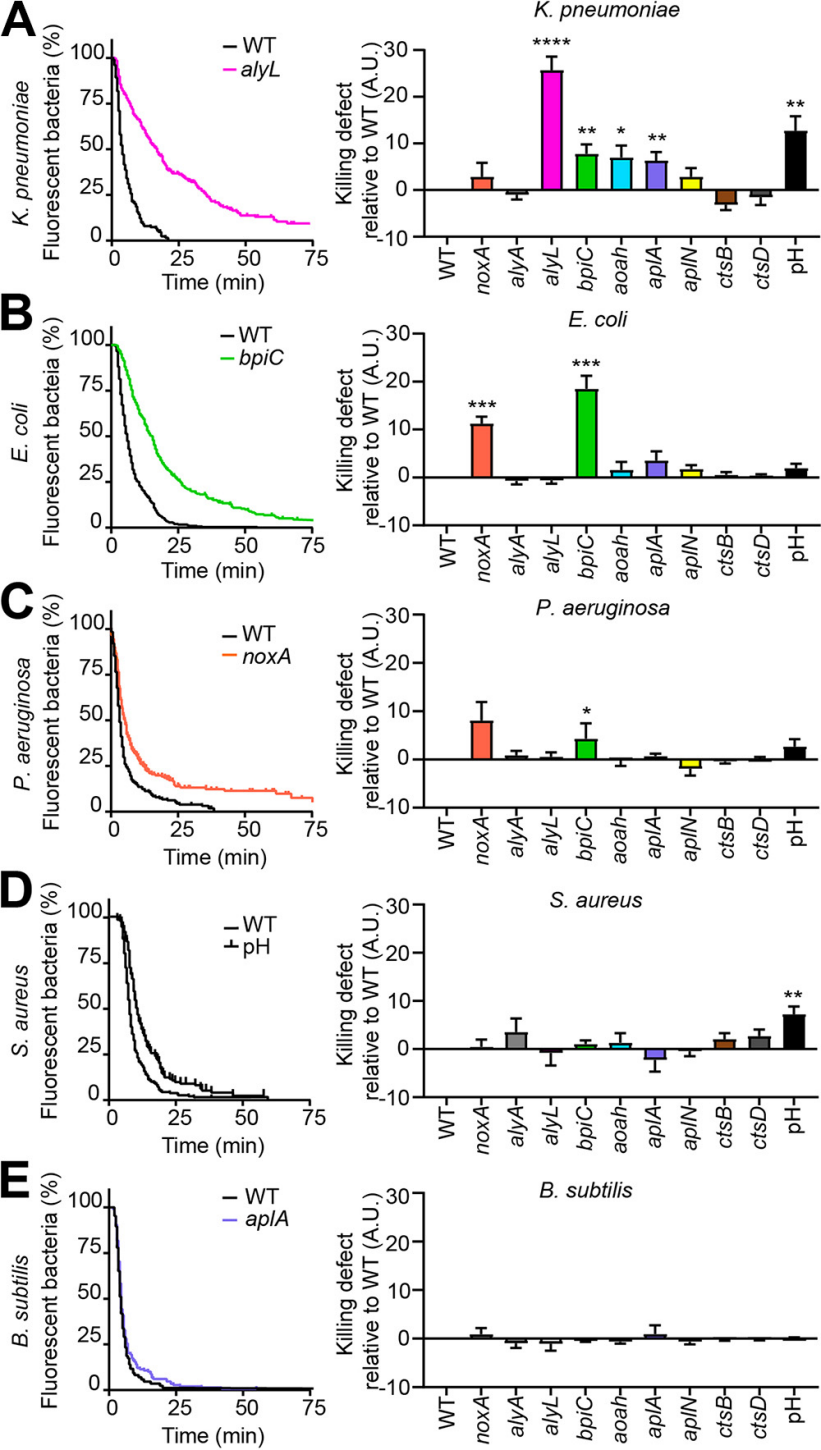
Role of effector proteins and pH in intracellular bacterial killing. The ability of a collection of D. discoideum mutant cells to kill different bacteria was assessed as shown in Fig. 1. NoxA, AlyA, AlyL, BpiC, Aoah, AplA, AplN, CtsB, and CtsD are putative effector proteins with different proposed modes of action. The role of the acidic phagosomal pH was tested by adding 40 mM NH_4_Cl during the experiment to the medium to raise the phagosomal pH. A.U., arbitrary units. (A, left panel) Percentage of fluorescent K. pneumoniae in WT and *alyL* KO mutant cells as a function of time after ingestion (*n* = 28). (Right panel) Defects in intracellular killing of K. pneumoniae were determined for each D. discoideum mutant and for the increase in pH (NH_4_Cl) as described in Fig. 1C. Values are means ± SEM. *, *P* < 0.05, **, *P* < 0.005, ***, *P* < 0.0005, and ****, *P* < 0.0001, Mann-Whitney test. Numbers of experiments (*n*) are as follows: *noxA*, *n* = 9; *alyA*, *n* = 5; *aplN*, *n* = 6; *alyL*, *n* = 28; *bpiC*, *n* = 16; *aoah*, *n* = 13; *aplA*, *n* = 8; *ctsB*, *n* = 7; *ctsD*, *n* = 11; pH, *n* = 8. (B to E) The intracellular killing of E. coli (B), P. aeruginosa (C), S. aureus (D), and B. subtilis (E) was determined in WT and mutant D. discoideum cells as described in panel A. For each bacterium, killing in WT cells and in the most severely altered mutant is shown on the left panel. Numbers of experiments for E. coli: *alyA*, *alyL*, and pH, *n* = 5; *aplN*, *aoah*, *aplA*, *ctsB*, and *ctsD*, *n* = 6; and *noxA* and *bpiC*, *n* = 11. Numbers of experiments for P. aeruginosa: *noxA*, *n* = 8; *alyA*, *alyL*, *aoah*, *aplA*, and pH, *n* = 5; and *aplN*, *bpiC*, *ctsB*, and *ctsD*, *n* = 6. Numbers of experiments for S. aureus: *noxA*, *alyL*, *bpiC*, *aoah*, *aplA*, and pH, *n* = 5; *ctsB*, *n* = 11; and *aplN*, *alyA*, and *ctsD*, *n* = 8. Numbers of experiments for B. subtilis: *noxA*, *alyA*, *alyL*, *bpiC*, *aoah*, *aplA*, and pH, *n* = 5; and *aplN*, *ctsB*, and *ctsD*, *n* = 6.

We then studied nine putative effectors of bacterial killing: NoxA, AlyA, AlyL, BpiC, Aoah, AplA, AplN, CtsB, and CtsD. For each gene, at least two independent KO cell lines were generated and analyzed in parallel with similar results. After confirming that all KO mutants were capable of ingesting fluorescent bacteria, we monitored the intracellular killing of bacteria as described in Fig. 1. Genetic inactivation of *alyL*, *bpiC*, *aoah*, or *aplA* slowed down intracellular killing of ingested K. pneumoniae (Fig. 2A). *noxA* KO and *bpiC* KO cells killed ingested E. coli cells more slowly than WT cells (Fig. 2B). Likewise, ingested P. aeruginosa cells were also killed more slowly in *noxA* KO and *bpiC* KO cells than in WT cells, but the variability of the experiments was higher, and the statistical significance of these results is questionable (Fig. 2C). The kinetics of intracellular killing of S. aureus and B. subtilis were unaffected in the nine mutants tested (Fig. 2D and E).

We tested the importance of phagosomal acidity by adding NH_4_Cl during the experiment, a procedure shown to significantly increase phagosomal pH (24). An increase in phagosomal pH slowed down the killing of K. pneumoniae and S. aureus (Fig. 2A and D), but had no significant effect on the killing of E. coli, P. aeruginosa, and B. subtilis (Fig. 2B, C, and E). These results show that the efficient intracellular killing of different bacteria requires largely different bactericidal mechanisms.

### Functional relationships between Kil1, Kil2, AlyL, Aoah, and BpiC during intracellular killing of K. pneumoniae.

**FIG 3:**
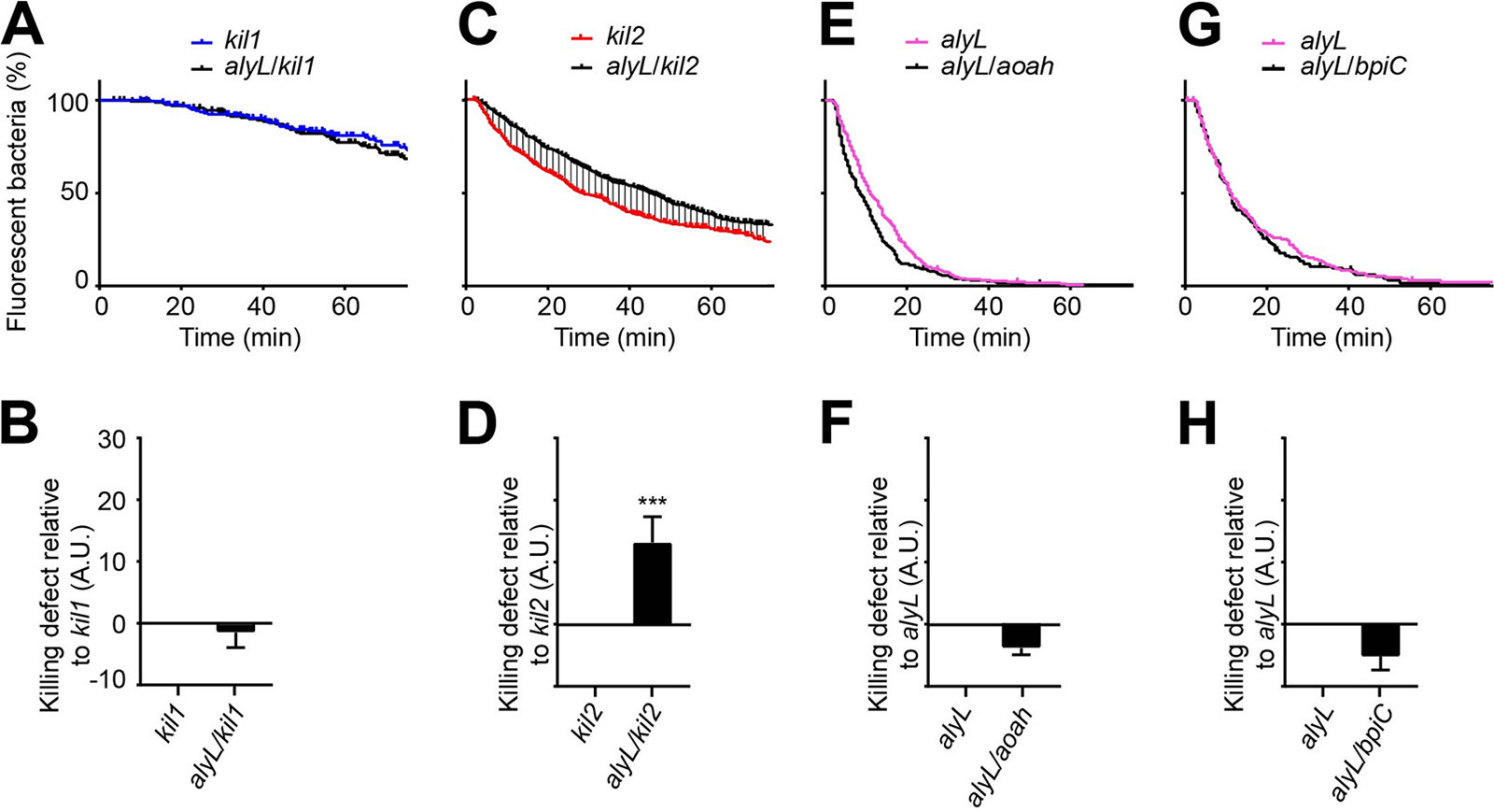
Functional relationships between Kil1, Kil2, AlyL, Aoah, and BpiC. The intracellular killing of K. pneumoniae was assessed in single and double KO mutants as shown in Fig. 1. (A and B) *kil1* KO was compared with *alyL kil1* double KO (*n* = 5), (C and D) *kil2* KO with *alyL kil2* double KO (*n* = 11), (E and F) *alyL* KO with *alyL aoah* double KO (*n* = 8), and (G and H) *alyL* KO with *alyL bpiC* double KO (*n* = 5). ***, *P* < 0.0005, Mann-Whitney test. Killing of ingested K. pneumoniae is slower in *alyL kil2* double KO cells than in *kil2* KO D. discoideum cells, indicating that AlyL is still functional in *kil2* KO cells (as its genetic inactivation further slows down killing of K. pneumoniae). A.U., arbitrary units.

As AlyL was quantitatively the most important effector in the killing of ingested K. pneumoniae, we attempted to delineate better its mechanism of action. In order to determine the functional relationships between AlyL and Kil1 and between AlyL and Kil2, we generated and analyzed *alyL kil1* and *alyL kil2* double KO mutants (Fig. 3). *alyL kil1* double KO cells exhibited no additional defect compared to *kil1* KO cells (Fig. 3A and B). On the contrary, *alyL kil2* double KO cells killed ingested K. pneumoniae slower than *kil2* KO cells (Fig. 3C and D). These results indicate that AlyL is functionally inactive in *kil1* KO cells, but still active in *kil2* KO cells.

We used the same strategy to assess the functional relationships of AlyL with BpiC and Aoah, two other gene products required for the killing of K. pneumoniae and expected to target the bacterial cell wall. We generated *alyL aoah* and *alyL bpiC* double KO mutants and assessed their ability to kill ingested K. pneumoniae. Neither *alyL aoah* nor *alyL bpiC* double KO mutants had an additional defect compared to *alyL* KO cells (Fig. 3E to H). These results indicate that in the absence of AlyL, Aoah, and BpiC are not active against K. pneumoniae.

### A distinct antibacterial region in AlyL.

**FIG 4:**
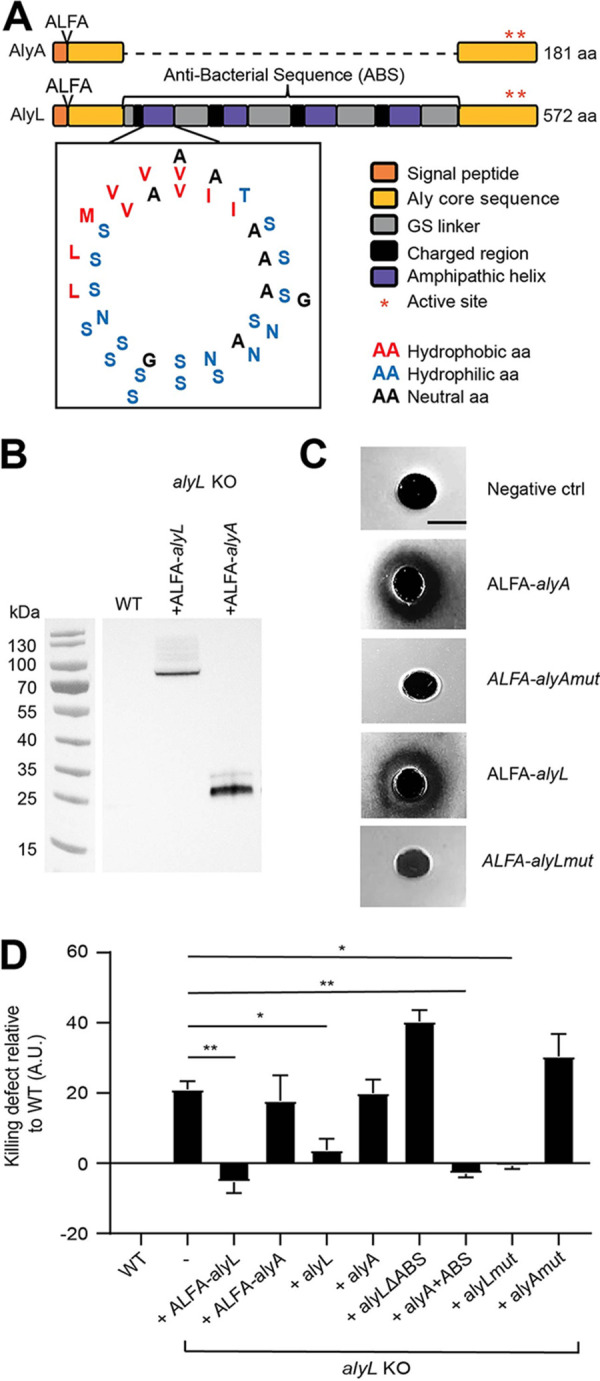
The ABS region of AlyL is responsible for its antibacterial activity against K. pneumoniae. (A) The sequences of AlyA and AlyL were aligned on their N-terminal 84 and C-terminal 94 residues. Between these two conserved regions, AlyL possesses a 394-residue region containing four repeated motifs each composed of about 10 mostly positively charged residues followed by 40 residues exhibiting one hydrophobic residue (red) every 3 to 4 residues (blue). The four repeated motifs are separated by poly(GS) flexible linkers (gray). Each of the four repeated motifs can be modeled as an amphipathic alpha-helix using the pepwheel program on EMBOSS. All the hydrophilic residues cluster on one side of the helix and the hydrophobic residues on the other side. The position where the ALFA tag was inserted is indicated at the N-terminal end. (B) ALFA-AlyL and ALFA-AlyA were overexpressed in *alyL* KO cells and detected in cellular lysates by Western blotting using a recombinant antibody against the ALFA tag. WT cells were used as negative control. (C) Purified ALFA-AlyL, ALFA-AlyA, ALFA-AlyAmut, and ALFA-AlyLmut proteins were deposited on an agarose plate containing cell wall extracts from *M. lysodeikticus*. Purified ALFA-tagged lysozymes digested the bacterial cell wall and formed a cleared zone, but enzymatically inactive mutant proteins did not. WT cells were used as the negative control (ctrl). (D) Intracellular killing of ingested K. pneumoniae was determined as shown in Fig. 1 in WT cells, *alyL* KO cells, and *alyL* KO cells overexpressing ALFA-AlyL, ALFA-AlyA, AlyL, AlyA, AlyL devoid of its ABS region (*alyL* ΔABS), AlyA with the AlyL ABS region added (*alyA*+ABS), or enzymatically inactive AlyA (AlyAmut) or AlyL (AlyLmut). Overexpression of AlyL (ALFA-tagged or native protein) in *alyL* KO D. discoideum restored rapid killing of ingested K. pneumoniae, while overexpression of AlyA (ALFA-tagged or native protein) did not have a measurable effect. AlyL missing its putative antibacterial region did not accelerate K. pneumoniae killing in *alyL* KO cells, but AlyA containing the antibacterial region of AlyL did. Values are means ± SEM. *, *P* < 0.05, and **, *P* < 0.005, Mann-Whitney test. *n* = 5 for overexpression of ALFA-*alyL*, ALFA-*alyA*, *alyL*, *alyL* ΔABS, and *alyA+*ABS, *n* = 11 for overexpression of *alyA*, and *n* = 4 for overexpression of *alyA*mut and *alyL*mut. A.U., arbitrary units.

The D. discoideum genome contains 22 genes encoding lysozymes from four different types (10). AlyA and AlyL belong to the Aly-type family and exhibit a high sequence identity at their N- and C-terminal regions, but AlyL contains an additional region compared to AlyA (Fig. 4A). The specific antibacterial function of AlyL may thus be due to its putative lysozyme activity or its additional region.

We first measured the whole cellular lysozyme enzymatic activity in *alyA* and *alyL* KO cells (see Fig. S3A in the supplemental material). In newly generated *alyA* KO cells, the total lysozyme activity decreased to about 25% of that in WT cells (Fig. S3B). Over a period of a few weeks in culture, the cellular lysozyme activity gradually increased and finally stabilized at a value corresponding to approximately 60% of WT cells (Fig. S3B). These observations are in accordance with a previous report, in which a genetic inactivation of AlyA resulted in a decrease of the cellular lysozyme activity of approximately 50% (14). The lysozyme activity measured in *alyL* KO cells was the same as that in WT cells (Fig. S3C), suggesting that AlyL does not contribute significantly to the total cellular lysozyme activity. In newly generated KO cells, the disruption of *alyL* but not *alyA* slowed the intracellular killing of K. pneumoniae (Fig. S3D and E). Overall, it appears that the decreased killing of ingested K. pneumoniae cells in *alyL* KO cells does not result from a decrease in the total cellular lysozyme enzymatic activity.

10.1128/mBio.03169-20.4 FIG S3 Related to Fig. 4. Cellular lysozyme activity and destruction of K. pneumoniae bacteria in *alyA* and *alyL* KO cells. (A) Lysates from WT or mutant cells were deposited on an agarose plate containing cell wall extracts from *M. lysodeikticus*. Lysozyme in the lysate digested the bacterial cell wall and formed a cleared zone, the size of which decreased when the lysozyme activity in the lysate was lower. The lysozyme activity of each lysate was calculated by comparison with serial dilutions of the WT lysate. (B) The lysozyme activity was determined in freshly generated *alyA* KO cells and in cells passed for longer periods of time. The first passage corresponds to expansion of original clones from 96-well plates. Note that it took approximately 5 weeks (cells passed 10 times) to identify mutant cells and a few more weeks to amplify them enough to freeze them. (C) The lysozyme activity was measured in lysates from *alyL* KO cells as described for panel B. (D and E) Intracellular destruction of K. pneumoniae was determined in freshly generated *alyA* KO or *alyL* KO cells (cells passed less than 20 times [p < 20]). Destruction of K. pneumoniae was slower in *alyL* KO cells than in WT cells. Download FIG S3, TIF file, 0.7 MB. Copyright © 2021 Jauslin et al. 2021 Jauslin et al. https://creativecommons.org/licenses/by/4.0/ This content is distributed under the terms of the Creative Commons Attribution 4.0 International license.

To test whether AlyL has an enzymatic activity, we expressed in *alyL* KO cells recombinant AlyA or AlyL proteins with an ALFA tag at their N terminus (Fig. 4A). The expression of each construct was verified by Western blotting using a recombinant antibody against the ALFA tag (Fig. 4B). ALFA-AlyA and ALFA-AlyL migrated with apparent molecular masses of approximately 25 and 80 kDa, respectively (Fig. 4B). We then purified the two proteins by immunoprecipitation and assessed their lysozyme activity. Both purified AlyA and AlyL exhibited lysozyme activity (Fig. 4C). We also expressed and purified ALFA-tagged mutants of AlyA (ALFA-AlyAmut) and AlyL (ALFA-AlyLmut), in which the presumptive catalytic site was mutated (E_100_→Q and D_109_→C for AlyA and E496→Q and D505→C for AlyL), and observed that this mutation indeed destroyed the lysozyme activity of purified AlyA and AlyL (Fig. 4C). We then measured the kinetics of K. pneumoniae killing in *alyL* KO cells overexpressing ALFA-AlyA and ALFA-AlyL. Fast killing of bacteria was restored upon expression of ALFA-AlyL, while overexpression of ALFA-AlyA did not have an effect (Fig. 4D). Similarly, expression of untagged AlyL restored fast killing in *alyL* KO cells, while expression of AlyA did not (Fig. 4D). Remarkably, the same observation was made when enzymatically inactive AlyL (AlyLmut) and AlyA (AlyAmut) were expressed: expression of AlyLmut restored fast killing in *alyL* KO cells, but expression of AlyAmut did not (Fig. 4D). Together these observations indicated that both AlyA and AlyL have a lysozyme enzymatic activity, but suggested that the contribution of AlyL to the total cellular lysozyme activity was negligible. Remarkably, only AlyL was involved in killing of K. pneumoniae in phagosomes, and the role of AlyL in killing did not require its lysozyme activity.

AlyL could in principle be required to establish and maintain the proper organization of the phagocytic pathway. To test this hypothesis, we verified whether loss of AlyL caused a general disruption of the phagocytic pathway: phagocytosis and macropinocytosis rates were indistinguishable in WT and *alyL* KO cells (see Fig. S4A and B in the supplemental material), as were the kinetics of phagosomal proteolysis (Fig. S4C), the acidification of phagosomes (Fig. S4D), and the general morphology of the endosomal pathway (Fig. S4E).

10.1128/mBio.03169-20.5 FIG S4 Related to Fig. 4. Genetic inactivation of *alyL* did not alter the organization of the endocytic pathway. (A) Phagocytosis of latex beads was as efficient in *alyL* KO cells and in WT cells. (B) Fluid-phase uptake of dextran was as efficient in *alyL* KO cells and in WT cells (mean ± SEM; *n* = 10). (C) Proteolytic activity in maturing phagosomes was identical in *alyL* KO cells and in WT cells (mean ± SEM; *n* = 3 beads). (D) Acidification of phagosomes was identical in *alyL* KO cells and in WT cells (mean ± SEM; *n* = 4 beads). (E) Immunofluorescence staining of WT and *alyL* KO cells with an anti-H^+^-ATPase antibody and an anti-p80 antibody. Lysosomes contain both H^+^-ATPase and p80 (arrowheads). Post-lysosomes contain p80 but no H^+^-ATPase (arrows). Contractile vacuoles contain H^+^-ATPase but no p80 (pinheads). No difference was observed between WT and *alyL* KO cells. Scale bar, 2 μm. A.U., arbitrary units. Download FIG S4, TIF file, 1.5 MB. Copyright © 2021 Jauslin et al. 2021 Jauslin et al. https://creativecommons.org/licenses/by/4.0/ This content is distributed under the terms of the Creative Commons Attribution 4.0 International license.

Finally, the additional 394-residue region found in AlyL but not AlyA could account for the specific antibacterial activity of AlyL. Upon close examination, we noticed that this region contains four conserved motifs separated by linkers rich in serine and glycine residues (see Fig. S5 in the supplemental material). Each of the four repeated motifs contains a charged region followed by a putative amphipathic alpha-helix: assuming an alpha-helical structure, hydrophobic residues cluster on one side of the helical wheel, while hydrophilic residues are found on the other side (Fig. 4A). In the remaining part of this article, this region is referred to as the putative AlyL antibacterial sequence (ABS). To assess the putative role of the ABS region, we constructed several lysozyme mutants and tested their ability to restore efficient bacterial killing in *alyL* KO cells. Expression of AlyL lacking the ABS region (AlyL ΔABS) did not restore efficient killing of K. pneumoniae in *alyL* KO cells, but expression of a chimeric AlyA carrying the AlyL ABS region (AlyA+ABS) did (Fig. 4D). These results demonstrated that the ABS region found in AlyL is responsible for its antibacterial activity against K. pneumoniae.

10.1128/mBio.03169-20.6 FIG S5 Related to Fig. 4. Primary structure of AlyA and AlyL. (A) The sequences of AlyA and AlyL were compared using ClustalW. AlyA and AlyL are highly homologous over a region spanning their first 84 and their last 94 residues. Between these two conserved regions, AlyL presents a 394-residue sequence (referred to as the antibacterial sequence [ABS]) containing four highly conserved motifs, each composed of about 10 positively charged amino acids followed by around 40 residues alternating one hydrophobic residue (red) every 3 to 4 hydrophilic residues (blue). The four repeated motifs are separated by poly(GS) linkers (gray). (B) Comparison of the four motifs present in the AlyL ABS. Download FIG S5, TIF file, 0.8 MB. Copyright © 2021 Jauslin et al. 2021 Jauslin et al. https://creativecommons.org/licenses/by/4.0/ This content is distributed under the terms of the Creative Commons Attribution 4.0 International license.

## DISCUSSION

In this study we used D. discoideum as a genetically tractable model phagocytic cell to study mechanisms of intracellular killing of bacteria.

Our first observation is that when we tested nine phagocytes depleted of a single putative effector, we observed a defect in intracellular bacterial killing for five of them (Aoah, NoxA, BpiC, AlyL, and AplA). Aoah is the only putative acyloxyacyl hydrolase that can be identified in the D. discoideum genome. On the contrary, all the other effectors tested belong to families of proteins with potentially overlapping functions: the D. discoideum genome encodes three Nox proteins, three Bpi proteins with a signal peptide, at least four annotated cathepsins, 17 Apl precursor proteins potentially giving rise to 33 amoebapore-like peptides, and 22 lysozymes, including 7 Aly proteins. One may have expected that functional redundancy within each family (e.g., one lysozyme compensating for the loss of another) or between families (e.g., Nox activity compensating for the loss of a lysozyme) would mask the effects of inactivating a single gene, but this was clearly not always the case. For example, it is remarkable that despite the existence of 22 lysozymes, the genetic inactivation of AlyL significantly slowed down the intracellular killing of K. pneumoniae. Similarly, genetic inactivation of AplA delayed killing of K. pneumoniae despite the existence of 16 other Apl precursor proteins. Some of this specificity may reflect differential expression of different genes. It was for example shown that NoxB and NoxC are only expressed during multicellular development of D. discoideum and not in unicellular amoebae (11). However, in larger protein families such as that of the Apl proteins, RNA sequencing experiments clearly indicate that multiple members of the family are expressed in unicellular amoebae (25). Two main explanations can be envisaged to account for this low degree of redundancy. The first explanation proposes that efficient killing of at least some bacteria (K. pneumoniae and E. coli) requires the full arsenal of intracellular killing mechanisms, and consequently, the loss of any single gene product can delay bacterial killing. The second explanation proposes that despite their similarities, distinct gene products act differently on different bacteria. In other words, AplA may be the only D. discoideum Apl capable of permeabilizing K. pneumoniae and AlyL the only lysozyme capable of lysing K. pneumoniae, inside the amoebae.

Our second observation stems from the detailed analysis of mutant phenotypes and also leads us to propose that different mechanisms are required to kill different bacteria in phagosomes. Indeed, the role and relative importance of the different gene products analyzed varied substantially when different bacteria were studied. Kil1 and Kil2 were both required for efficient killing of K. pneumoniae and of E. coli. However, the intracellular killing of these two enterobacteria necessitated largely different effectors: AlyL, BpiC, Aoah, and AplA for K. pneumoniae and NoxA and BpiC for E. coli. Killing of P. aeruginosa apparently mobilized a set of gene products (Kil2, BpiC, and NoxA) most similar to that required for E. coli killing, but significantly different from it. (Kil1 is required for efficient killing of E. coli but not P. aeruginosa.) Efficient killing of S. aureus required only Kil2. Remarkably, none of the mutants analyzed showed any defect in the killing of B. subtilis. B. subtilis may be much easier to kill than the other bacteria studied, and consequently, due to a high degree of redundancy, no single gene product would be essential for efficient killing of B. subtilis. Alternatively, the killing of B. subtilis may mobilize entirely different molecular mechanisms, not analyzed in this study. The two models are not mutually exclusive, and only a large number of additional experiments will allow the evaluation of their relevance.

Our third set of observations defines the role of AlyL in the killing of K. pneumoniae. Characterization of double mutants suggested that AlyL, Aoah, and BpiC have overlapping functions. It is known that Aoah and BpiC target bacterial LPS, while lysozymes can digest peptidoglycans and alter membrane integrity (3, 19, 26). A simple interpretation of our observations is that Aoah and BpiC disrupt the protective LPS layer, thereby allowing AlyL to gain access to the peptidoglycan layer and to the bacterial cytoplasmic membrane.

AlyA and AlyL have previously been characterized by functional genetics (14, 15). Our results confirmed the observation that AlyA is the major contributor to the cellular lysozyme activity. On the contrary, genetic inactivation of AlyL produced no detectable decrease in the total cellular lysozyme activity, indicating that its contribution to the total cellular lysozyme activity was negligible. Hence, it came as a surprise to find that AlyA was dispensable for efficient killing of all bacteria analyzed, while AlyL was the most important of the examined effectors for the killing of K. pneumoniae. This was due to the presence of a supplementary element in AlyL, a large flexible loop exhibiting four amphipathic helical motifs. Amphipathic helices have been shown to interact with membranes, insert in lipid bilayers and form transmembrane pores. This is a structural element often observed in antimicrobial peptides or proteins (27, 28). Accordingly, the extended ABS loop may confer on AlyL an additional ability to permeabilize bacterial membranes. At the conceptual level, there is a striking similarity between these observations and previous reports on the mode of action of vertebrate lysozymes. Indeed, elegant experiments have shown that the lysozyme activity of the murine LysM is not required for its bacterial killing activity *in vitro* or *in vivo* (29). Similarly, the membrane-permeabilizing activity of hen egg lysozyme accounts for its antibacterial activity and may implicate a region distinct from its catalytic center (30). This situation may constitute a remarkable example of convergent evolution in which both animal lysozymes and a D. discoideum lysozyme associate a lysozyme enzymatic domain with a membrane-permeabilizing region. The advantage obtained by combining two different activities (enzymatic and bacteriolytic) on a single protein remains to be established.

Together, the results presented in this study underline the limitations in our understanding of intracellular bacterial killing. First, most of our *a priori* predictions concerning the importance of individual gene products were at least partially contradicted by our results. Second, our results mostly shed light on the mechanisms underlying killing of K. pneumoniae, but even for this bacterium, the current list of identified effectors remains largely incomplete. Third, we have not identified a single mechanism involved in the killing of B. subtilis. Clearly, our knowledge about the mechanisms involved in the intracellular killing of bacteria is still largely fragmentary.

Finally, it should be stressed that throughout this study, killing of bacteria was evaluated by determining the extinction of fluorescent proteins in ingested bacteria. This is the only practical method allowing us to follow individual bacteria in phagosomes and to detect even minor delays in killing. Other methods can in principle be used to detect intracellular killing, such as determining the ability of individual bacteria to regrow, the permeabilization of the bacterial cell wall, or alterations of bacterial morphology. A recent study suggests that in mouse alveolar macrophages, Nox-dependent bacterial cell wall permeabilization precedes enzymatic bacterial degradation (31). If the techniques used in this study can be applied in D. discoideum, they would allow us to dissect more precisely the role of individual gene products at different stages of the process of bacterial killing and destruction.

## MATERIALS AND METHODS

### Cell culture and strains.

Dictyostelium discoideum cells were cultured in HL5 medium (32) at 21°C and subcultured twice a week to maintain a cellular density below 10^6^ cells/ml. The D. discoideum mutant strains used in this study were derived from the DH1-10 subclone (33) of the D. discoideum strain DH1 (34), referred to in this study as wild type (WT), and are described in the supplemental material.

Bacterial strains were grown overnight in LB medium at 37°C. The bacteria used were K. pneumoniae KpGe (35), E. coli REL606 (36), nonsporulating B. subtilis strain 36.1 (37), a fluorescent K. pneumoniae KpGe strain expressing yeast-enhanced GFP (yEGFP) (38), a flagellum-less B. subtilis strain expressing mCherry (38), S. aureus ATCC 29213 expressing enhanced green fluorescent protein (EGFP) using the plasmid pBSU101 (a kind gift from Paul O. Verhoeven [39]), and P. aeruginosa PT531 expressing GFP using the plasmid pIApX2, a kind gift of Ina Attrée (40). To generate fluorescent E. coli bacteria, the E. coli strain was transfected with a plasmid constitutively expressing codon‐optimized yeast‐enhanced GFP (yEGFP) and conferring resistance to kanamycin (38).

### Overexpression of *alyA* and *alyL* proteins.

The cDNA sequences of *alyA* and *alyL* fused (at the N-terminal end) to the ALFA tag sequence (SRLEEELRRRLTE) (41) were synthesized by Thermo Fisher Scientific and cloned into the G418-resistant prepSC3 vector as described previously (HindIII/KpnI) (42). The expression vectors were transfected into *alyL* KO cells, and transfected cells were selected using G418 at a concentration of 15 μg/ml. Overexpression of ALFA-AlyA and ALFA-AlyL proteins in individual clones was verified by Western blotting using AL626 anti-ALFA antibody (43) (Fig. 4B). The AL626 antibody used in this study was produced as a VHH-Fc_mouse_ miniantibody by the Geneva Antibody Facility of the University of Geneva (https://www.unige.ch/medecine/antibodies/).

### Detection of tagged proteins by Western blotting.

D. discoideum cells expressing ALFA-AlyA or ALFA-AlyL (1 × 10^6^) were pelleted and resuspended in 20 μl of reducing sample buffer (20.6% [wt/vol] sucrose, 100 mM Tris [pH 6.8], 10 mM EDTA, 0.1% [wt/vol] bromophenol blue, 4% [wt/vol] SDS, 6% [vol/vol] β-mercaptoethanol). Each sample was subjected to electrophoresis (200 V, 35 min) on a 4 to 20% acrylamide gel (Mini-PROTEAN TGX precast gel; Genscript 00655) and transferred to a nitrocellulose membrane using a dry transfer system for 7 min (iBlot gel transfer device; Invitrogen IB1001EU). The membranes were blocked for 2 h in phosphate-buffered saline (PBS)-Tween containing 7% (wt/vol) milk and then washed three times for 5 min in PBS-Tween. The membranes were incubated with AL626 overnight at 4°C and then washed three times for 5 min. The membranes were then incubated for 1 h in PBS-Tween containing horseradish peroxidase-coupled goat anti-mouse IgG (Bio-Rad no. 170-6516 [dilution of 1:3,000]) and washed twice for 5 min and once for 15 min in PBS-Tween. The signal was revealed by enhanced chemiluminescence (ECL) (Amersham Biosciences) using a PXi-4 gel imaging system (Syngene).

### Immunoprecipitation of tagged proteins.

A total of 1 × 10^9^ D. discoideum cells overexpressing ALFA-tagged proteins were pelleted, washed in PBS, and lysed in 10 ml of lysis buffer (PBS plus 0.5% [vol/vol] Triton X‐100 containing protease inhibitors (Thermo Fisher A32963) for 10 min at 4°C. Nuclei were pelleted, and the recovered supernatant was mixed with 50 μl of ALFA Selector PE resin (Nanotag Biotechnologies N1510) prewashed in PBS plus 0.1% (vol/vol) Tween 20 (41). After 1 h of incubation at 4°C on a wheel, the resin was recovered and washed 5 times with 1 ml of PBS plus 0.1% (vol/vol) Tween 20. Proteins attached to the resin were finally eluted with 100 μl of 0.1 M glycine-HCl (pH 3) containing protease inhibitors (Thermo Fisher A32963).

### Lysozyme activity.

Lysozyme activity was measured as described previously (44). Briefly, a 2-mm-thick layer of agarose (0.9%) containing 50 mM sodium acetate (pH 4.5) and 0.5 mg/ml lyophilized cell wall from Micrococcus lysodeikticus (M3770; Sigma-Aldrich) was poured into sterile petri dishes, and several 4-mm-diameter holes were made. D. discoideum cells were grown in suspension, and 10^8^ cells were centrifuged (1,500 × *g*, 10 min) and then washed once with 5 ml of PB buffer (defined below). Each pellet was resuspended in 10 times its volume of 10% acetic acid containing protease inhibitors (5 mg/ml iodoacetamide, 47 μM leupeptin, 1.5 μM aprotinin, 100 μM phenylmethylsulfonyl fluoride) and was rotated on a wheel at 4°C overnight. Finally, the cell extract was centrifuged in an Airfuge (150,000 × *g*, 4°C, 15 min), the supernatant was collected and deposited (20 μl) into the holes on an agarose-*Micrococcus* plate, and the plate was incubated at 37°C for 24 h. Alternatively, purified ALFA-AlyL, ALFA-AlyA, ALFA-AlyAmut, and ALFA-AlyLmut tagged proteins were deposited (20 μl) into the holes. Lysozyme activity created a clear halo around the holes. The relative lysozyme activity in mutant cells was assessed by comparing the halo diameter with that created by applying various dilutions of WT extracts. Hen egg white lysozyme (Sigma) served as a control.

### Intracellular killing of bacteria assessed by live microscopy.

The intracellular killing of fluorescent bacteria was measured as described previously (38). Briefly, fluorescent bacteria and 7 × 10^5^ D. discoideum cells were washed in phosphate buffer (PB: 2 mM Na_2_HPO_4_, 14.7 mM KH_2_PO_4_, pH 6.0) supplemented with 100 mM sorbitol (PB-sorbitol). Fluorescent bacteria were deposited in a glass-bottom well (μ-slide 8-well; IBIDI) and allowed to sediment for 5 min before the addition of D. discoideum cells. When using P. aeruginosa or E. coli, the slides were centrifuged after addition of the D. discoideum cells to ensure efficient sedimentation of bacteria and cells (250 × *g*, 10 min). When B. subtilis was used, bacteria were centrifuged at 250 × *g* for 10 min before and after addition of D. discoideum cells.

To test the effect of phagosomal pH on intracellular killing of bacteria, D. discoideum cells were washed and observed in PB-sorbitol supplemented with 40 mM NH_4_Cl. NH_4_Cl treatment has been shown to increase the pH in endosomal compartments (24).

To image the whole-cell volume at each time point, an image (bright field and with filters for GFP or mCherry) was taken in five successive focal planes with a step size of 3 μm every 30 s for 2 h with a Nikon eclipse Ti2 wide-field time-lapse microscope equipped with a DS-Qi2 camera. The NIS software was used to extract the images, and Fiji was used to compile and analyze movies. For each bacterium, time zero was the time it entered a D. discoideum cell and bacterial killing was indicated by the loss of its fluorescence. Intracellular killing of phagocytosed bacteria was calculated using the Kaplan-Meier estimator on Prism version 7.0.a GraphPad. In order to evaluate variability between independent experiments and to determine the significance of the observed differences, we calculated for each independent experiment the area under the curve (AUC) between 0 min and 75 min, subtracted the AUC of the control condition (WT cells [AUC_WT_]), and normalized the result to the maximum killing activity (100 × 75 − AUC_WT_). The detailed method used to calculate and represent graphically our observations is described in Fig. S1 in the supplemental material.

Additional information on the materials and methods used in our study can be found in Text S1 in the supplemental material.

10.1128/mBio.03169-20.1 TEXT S1 Supplemental materials and methods. Download Text S1, DOCX file, 0.03 MB. Copyright © 2021 Jauslin et al. 2021 Jauslin et al. https://creativecommons.org/licenses/by/4.0/ This content is distributed under the terms of the Creative Commons Attribution 4.0 International license.
