# Supplementary figures and images for: How Phagocytic Cells Kill Different Bacteria: a Quantitative Analysis Using Dictyostelium discoideum
Source: mBio. 2021 Feb 16;12(1):e03169-20. doi: 10.1128/mBio.03169-20 (PMC8545105; doi:10.1128/mBio.03169-20)

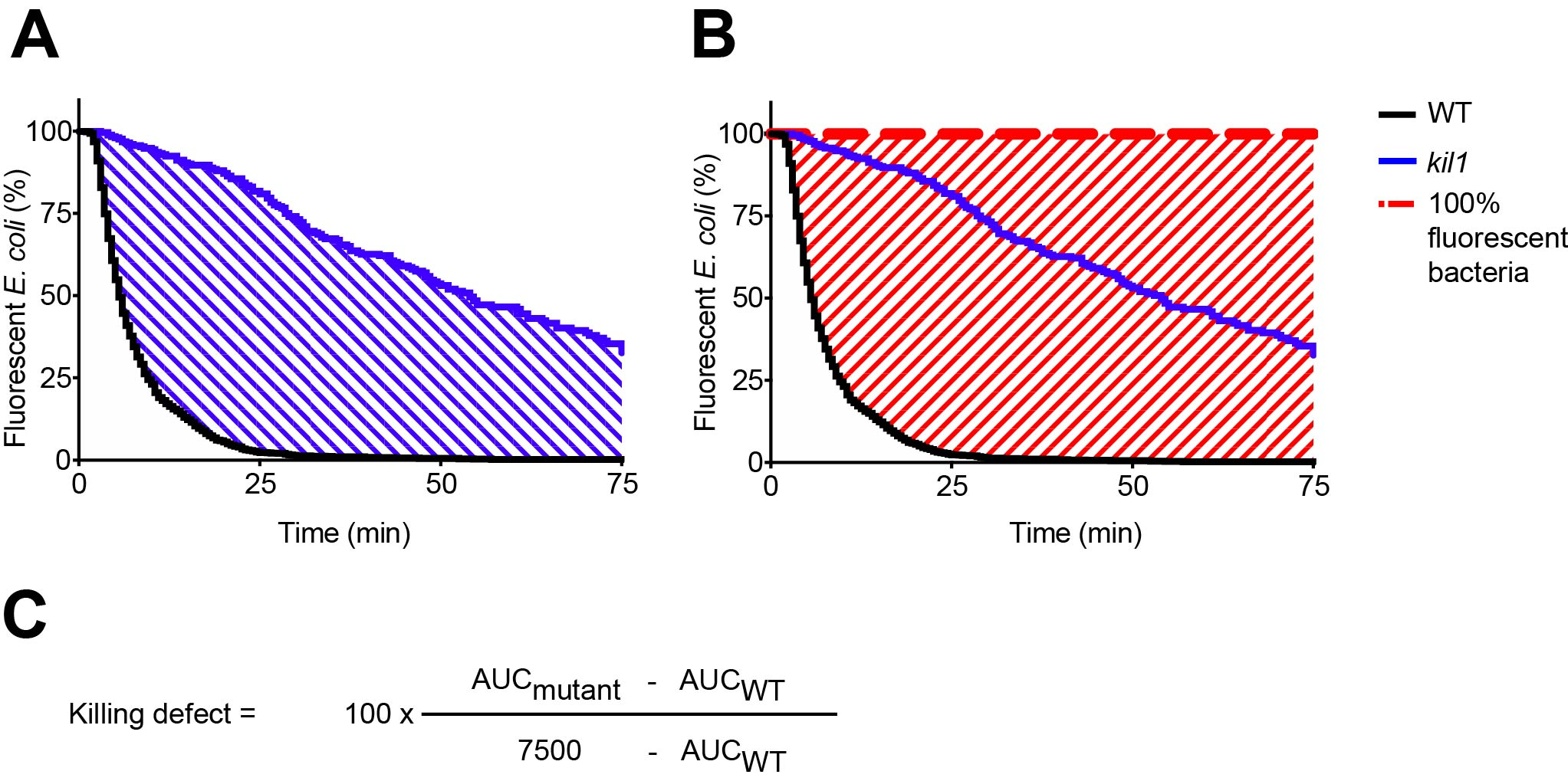

Supplement: FIG S1 [file mbio.03169-20-sf001.tif]

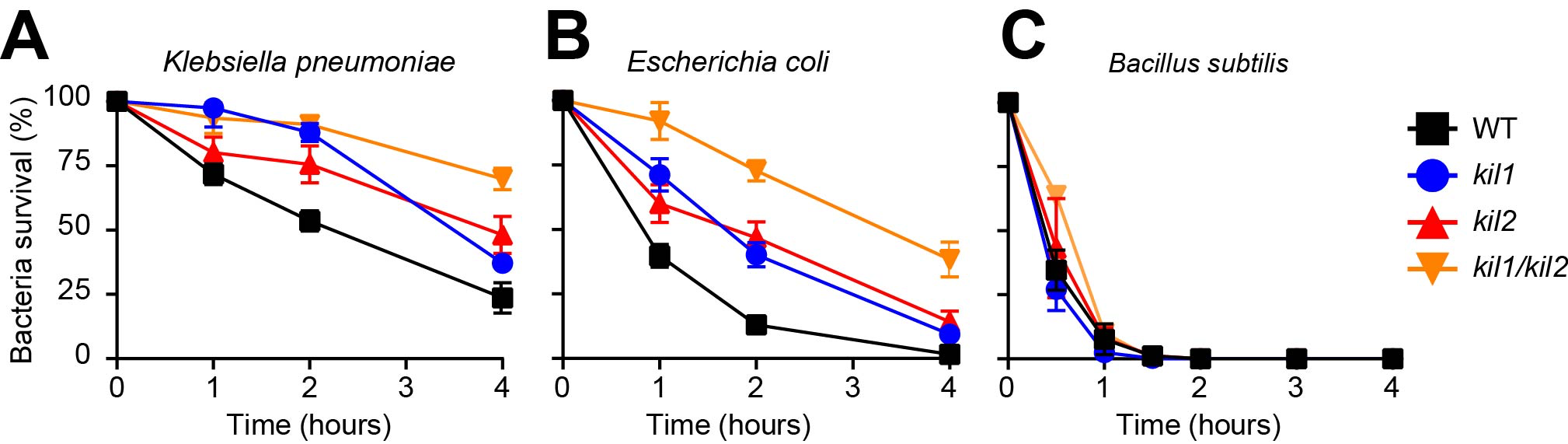

Supplement: FIG S2 [file mbio.03169-20-sf002.tif]

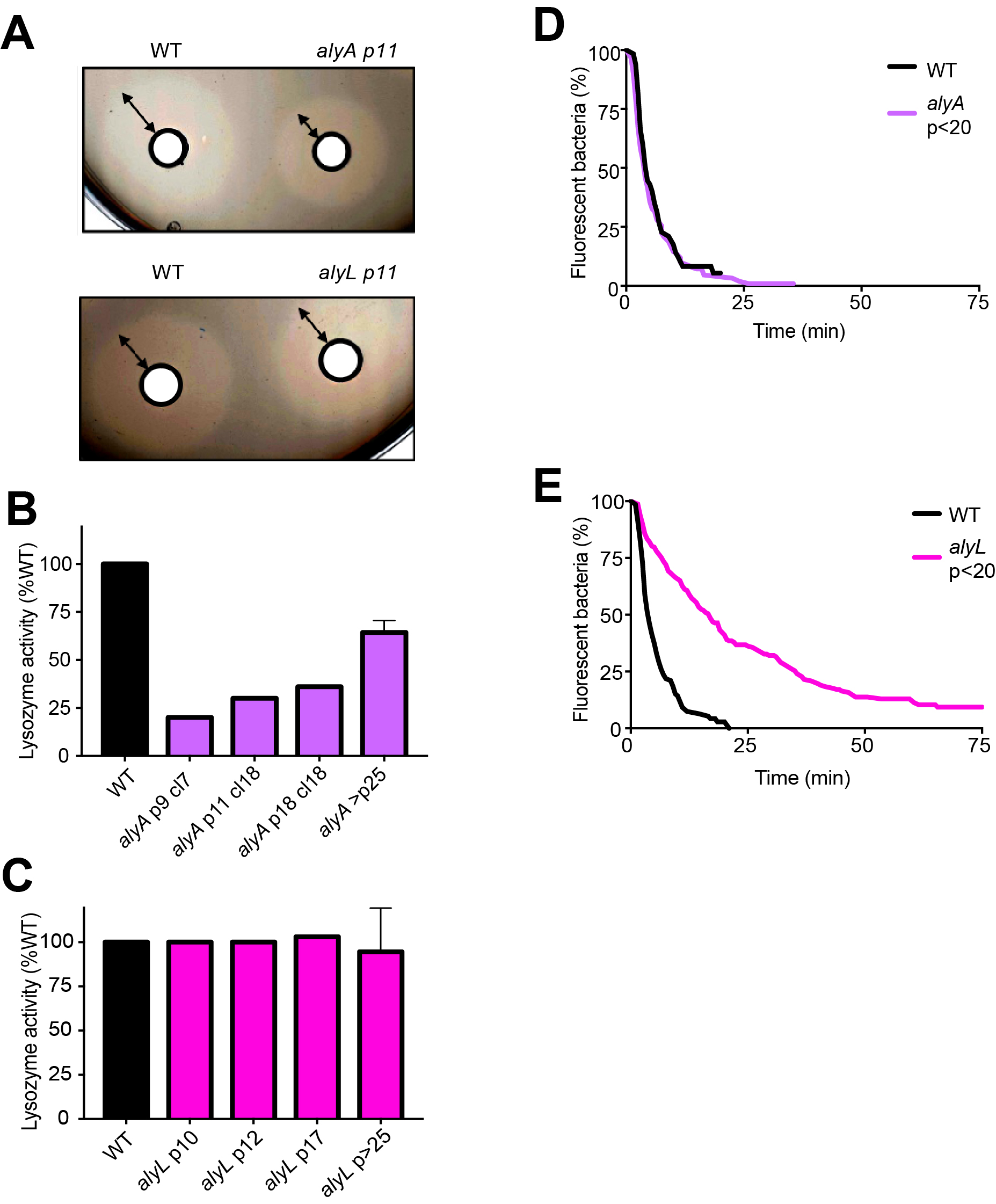

Supplement: FIG S3 [file mbio.03169-20-sf003.tif]

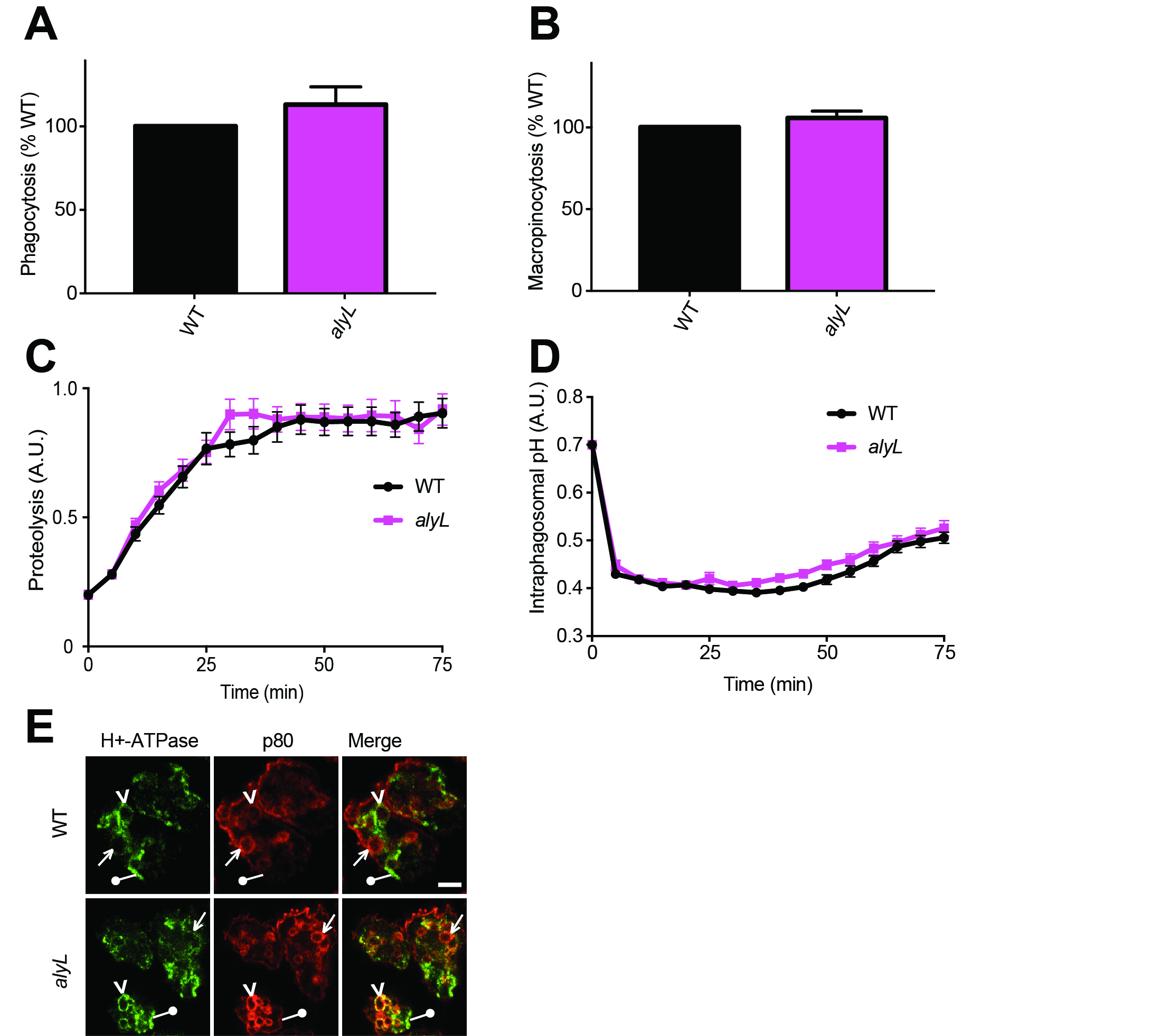

Supplement: FIG S4 [file mbio.03169-20-sf004.tif]

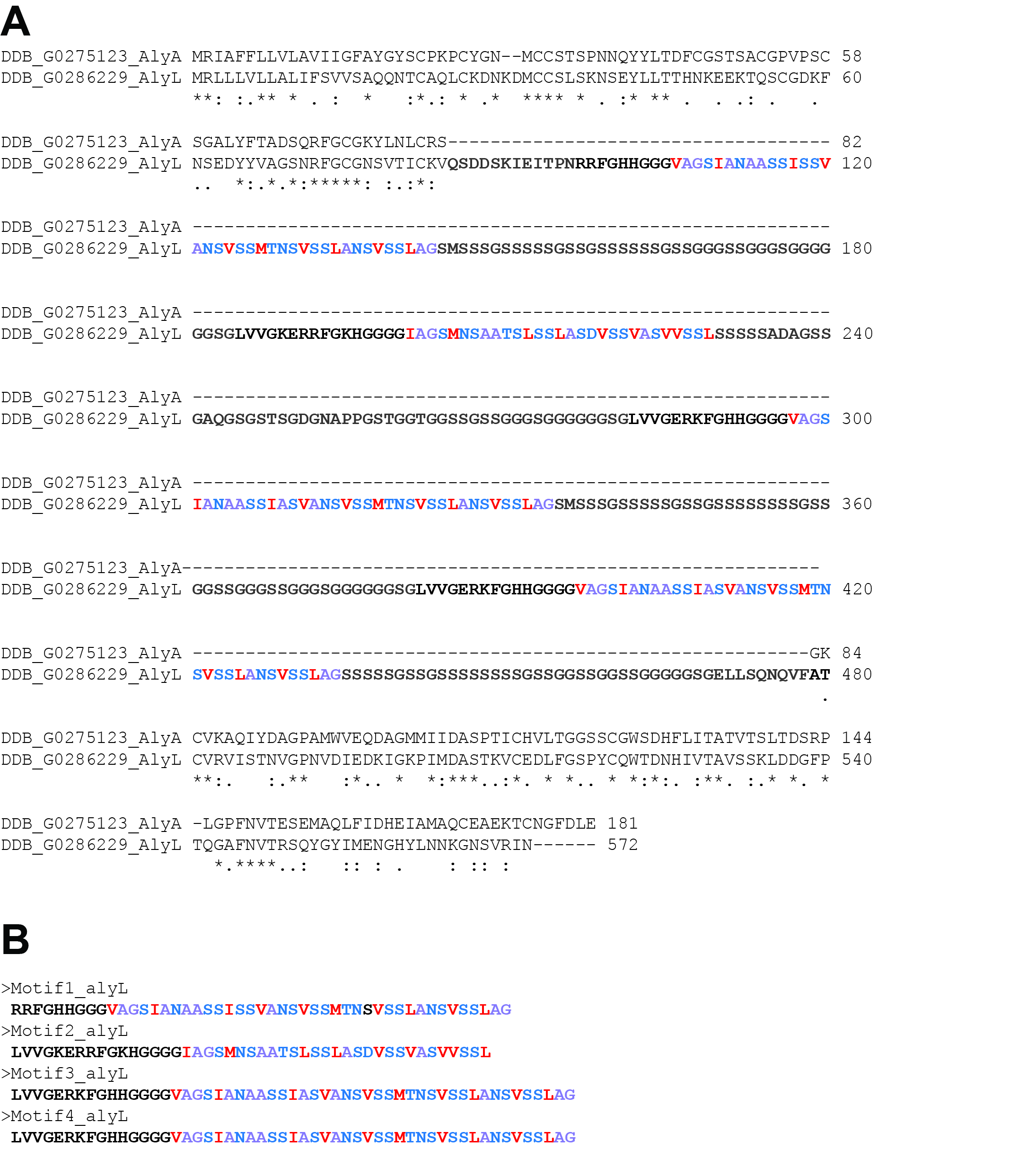

Supplement: FIG S5 [file mbio.03169-20-sf005.tif]
