# Supplementary material for: How Phagocytic Cells Kill Different Bacteria: a Quantitative Analysis Using Dictyostelium discoideum
Source: mBio. 2021 Feb 16;12(1):e03169-20. doi: 10.1128/mBio.03169-20 (PMC8545105; doi:10.1128/mBio.03169-20)
Supplement: TEXT S1 [file mbio.03169-20-s0001.docx]

**SUPPLEMENTAL Material and METHODS**

**Cell culture and strains**

The *kil1, kil2, alyL, alyA, aplA, aplN, noxA, aoah* and *bpiC* knockout (KO) strains were created by deleting a portion of the gene of interest in WT cells and replacing it with a blasticidin resistance (BSR) cassette. KO vectors were constructed by cloning homology regions into a pKOSG-blasticidin-resistant plasmid (1). Transfected cells were selected using blasticidin at a concentration of 10 µg/mL. Individual KO clones were screened by PCR (2). For each mutant at least two different clones were analyzed and displayed identical phenotypes.

The *ctsB* and *ctsD* KO cells were created using CRISPR/Cas9 as described previously (3). Briefly, sgRNA sequences for each gene was chosen using the website <http://www.rgenome.net/cas-designer/>. Then, the sgRNA was cloned into the pTM1285 plasmid using the Golden-gate assembly and the BpiI enzyme before transformation in TOP10 *E. coli*. Plasmids were purified using a maxiprep kit (Macherey-Nagel, #740574.25) and sequenced. *D. discoideum* cells (16 x 10^6^ cells in 400 μL) were electroporated with the pTM1285/sgRNA plasmid (20 μg) using a Bio-Rad Electroporator (Gene Pulser Xcell™ System) (4). Transfected *D. discoideum* cells were resuspended in 35 mL of HL5 medium. The next day, 15 μg/mL of G418 was added to the medium to selected plasmid-containing cells. After 6 days under selection, *D. discoideum* cells were cloned in the absence of G418 in 96-well plates with 3-fold dilutions in order to obtain individual clones of *D. discoideum* in some wells. To analyze individual clones, the genomic region of interest was amplified by PCR and sequenced. Two clones presenting different mutations were kept for further analysis. To obtain double-knockout mutants, the blasticidin-resistance cassette was excised by extrachromosomal expression of Cre (5).

**Killing of bacteria determined by counting colony-forming units**

The killing of bacteria was determined by counting colony-forming units as described previously (6). Briefly, 2 x 10^6^ *D. discoideum* cells were pelleted and washed twice in 1 mL PB buffer. 200 µL bacteria from a 14-hours overnight culture were pelleted and washed twice in PB, then diluted to OD_600_ = 0.2, and diluted to 10^-4^. 500 µL of washed *D. discoideum* and bacteria were co-incubated in horizontal Eppendorf tubes at 21 °C and shaken at 200 rpm. 10 µL of the suspension was collected at each timepoint (0, 0.5, 1, 2, 3 and 4 hours) and *D. discoideum* cells were lysed by successive addition of 40 µL sucrose 40% and 150 µL PB-saponin 0.5%. The bacteria were then plated on LB-agar plates, and bacterial colonies counted after an overnight incubation at 37 °C.

**Phagocytosis and macropinocytosis**

Phagocytosis and macropinocytosis were studied as described previously (6). In brief, phagocytosis and macropinocytosis were assessed by pelleting 3 x 10^5^ *D. discoideum* cells and incubating them in 750 µL HL5 medium containing 1 µm FITC-labeled latex beads (1 µL/mL) to assess phagocytosis or Alexa647-labeled dextran (Molecular Probes, Eugene, OR, USA) to assess macropinocytosis. Cells were incubated at 21°C in a shaken suspension for 20 min. After the incubation, cells were washed in ice-cold HL5 medium supplemented with 0.1% NaN_3_, and internalized fluorescence was measured by flow cytometry (Accuri). For each condition, mean fluorescence internalized by mutant cells was normalized to the fluorescence internalized by WT cells in the same experiment.

**Organization and function of endosomal pathways**

The pH and proteolytic activity of phagosomes was studied as described previously (7, 8). Briefly, 7 x 10^5^ *D. discoideum* cells were washed in PB-Sorbitol buffer and deposited on a glass slide (µ-slide 8-well, IBIDI).

To measure the proteolytic activity in phagosomes, cells were incubated with 3-µm carboxylated silica beads (Kisker Biotech; PSI‐3.0COOH) coupled with a green proteolysis-sensitive probe (DQ™ Green BSA; ThermoFisher D12050), and a red proteolysis-insensitive probe (Alexa 594-succinimidyl ester; ThermoFisher A20004).

To determine the pH of phagosomes, cells were incubated with 3-µm carboxylated silica beads (3 μm; Kisker Biotech; PSI‐3.0COOH) coupled with a pH-sensitive green fluorescent probe (FITC) and a pH-insensitive red fluorescent probe Alexa 594-succinimidyl ester (ThermoFisher A20004).

*D. discoideum* cells were imaged every 75 sec for 3 h with a Nikon eclipse Ti2 widefield time-lapse microscope equipped with a DS-Qi2 camera. To image the whole cell volume at each time point, an image (brightfield, GFP (to follow the proteolysis-sensitive probe), and RFP (to follow the proteolysis-insensitive probe) was taken in five successive focal planes with a step size of 3 µm. The NIS software was used to extract the images, and Fiji was used to compile and analyze movies. Time 0 is the time when the bead enters *D. discoideum.* The ratio of fluorescence (GFP/RFP) was calculated, normalized to time 0, and plotted on Graphpad.

To visualize endosomal compartments by immunofluorescence, *D. discoideum* cells (1 x 10^6^) were sedimented on a 22 x 22-mm glass coverslip (Menzel-Gläser) at room temperature in HL5 medium for 30 min. Cells were then fixed in 4% (w/v) paraformaldehyde and permeabilized in methanol at -20 °C for 3 min. The coverslip was then incubated with 200 µL primary antibody for 1 h at room temperature. Antibodies against Alexa647-coupled-p80 (H161, diluted 1:400) and vacuolar H^+^-ATPase (221-35-2, diluted 1:3) were diluted in PBS containing 0.2 % (w/v) BSA (PBS-BSA). Coverslips were then washed three times in PBS-BSA for 5 min and incubated in 200 µL Alexa-488-coupled goat anti-mouse immunoglobulin (diluted 1:300 in PBS-BSA, Life Technologies, #A11029) for 45 min. Finally, cells were washed three times in PBS-BSA for 5 min, then once in PBS, and mounted on 76 x 26-mm Menzel-Gläser slides with Möwiol (Hoechst) + 2.5 % (w/v) DABCO (Fluka, #33480). Pictures were taken using a Zeiss LSM800 confocal microscope with a 63x oil immersion objective.

**SUPPLEMENTAL REFERENCES**

1. Wiegand S, Kruse J, Gronemann S, Hammann C. 2011. Efficient generation of gene knockout plasmids for *Dictyostelium discoideum* using one-step cloning. Genomics 97:321-325.

2. Charette SJ, Cosson P. 2004. Preparation of genomic DNA from *Dictyostelium discoideum* for PCR analysis. Biotechniques 36:574-5.

3. Sekine R, Kawata T, Muramoto T. 2018. CRISPR/Cas9 mediated targeting of multiple genes in *Dictyostelium*. Scientific Reports 8:8471.

4. Alibaud L, Cosson P, Benghezal M. 2003. *Dictyostelium discoideum* transformation by oscillating electric field electroporation. BioTechniques 35:78-80.

5. Linkner J, Nordholz B, Junemann A, Winterhoff M, Faix J. 2012. Highly effective removal of floxed Blasticidin S resistance cassettes from *Dictyostelium discoideum* mutants by extrachromosomal expression of Cre. European Journal of Cell Biology 91:156-160.

6. Leiba J, Sabra A, Bodinier R, Marchetti A, Lima WC, Melotti A, Perrin J, Burdet F, Pagni M, Soldati T, Lelong E, Cosson P. 2017. Vps13F links bacterial recognition and intracellular killing in *Dictyostelium*. Cell Microbiol 19:e12722.

7. Sattler N, Monroy R, Soldati T. 2013. Quantitative analysis of phagocytosis and phagosome maturation. Methods in Molecular Biology 983:383-402.

8. Bodinier R, Leiba J, Sabra A, Jauslin TN, Lamrabet O, Guilhen C, Marchetti A, Iwade Y, Kawata T, Lima WC, Cosson P. 2020. LrrkA, a kinase with leucine-rich repeats, links folate sensing with Kil2 activity and intracellular killing. Cell Microbiol 22:e13129.
